# Supplementary material for: V-Domain Ig Suppressor of T Cell Activation (VISTA) Expression Is an Independent Prognostic Factor in Multiple Myeloma
Source: Cancers (Basel). 2021 May 6;13(9):2219. doi: 10.3390/cancers13092219 (PMC8124446; doi:10.3390/cancers13092219)
Supplement: Supplementary file 1 [file cancers-13-02219-s001.zip › cancers-1176054-supplementary.pdf]

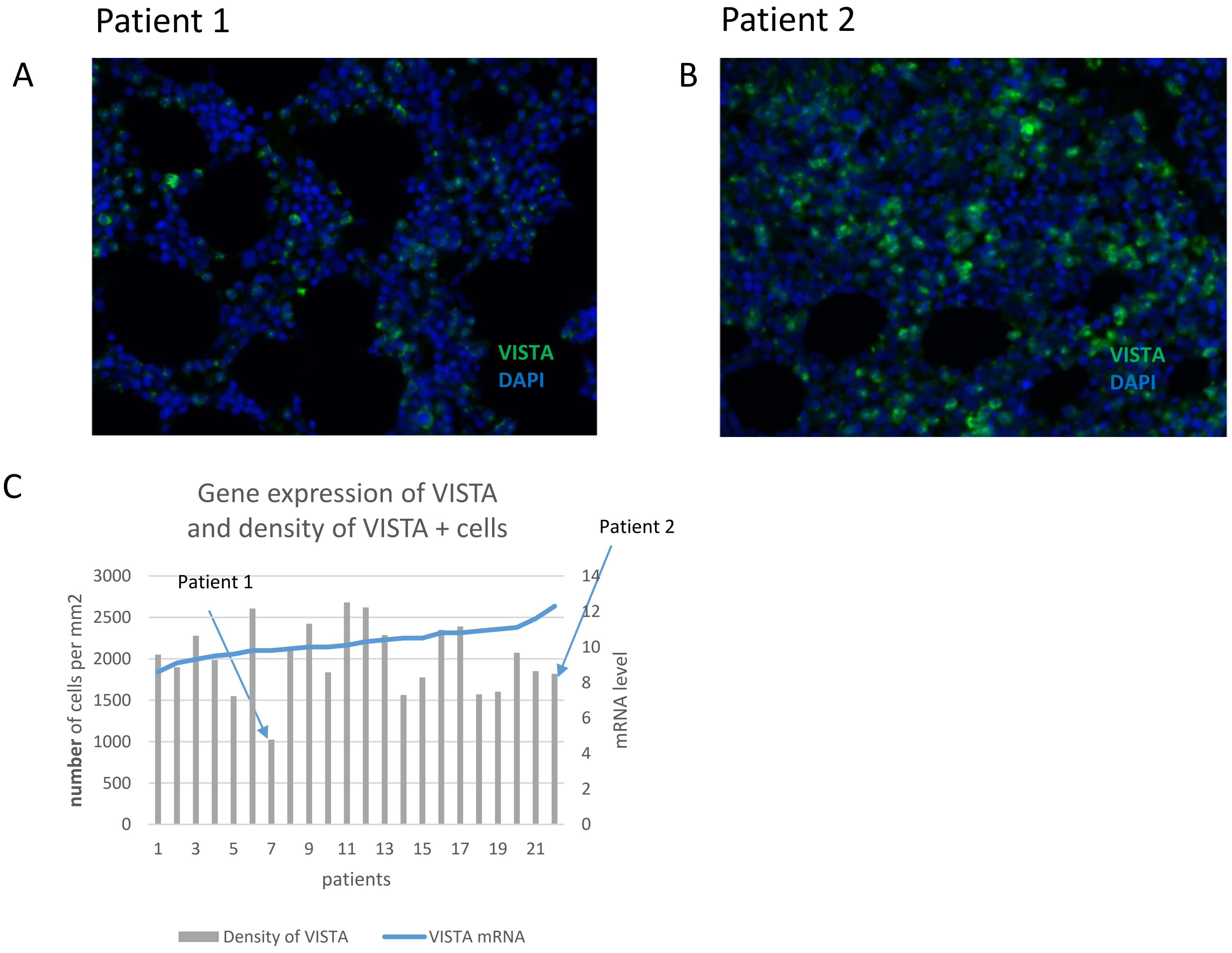

Supplementary figure 1: Density of VISTA+ cells does not concur with VISTA gene expression in MM.

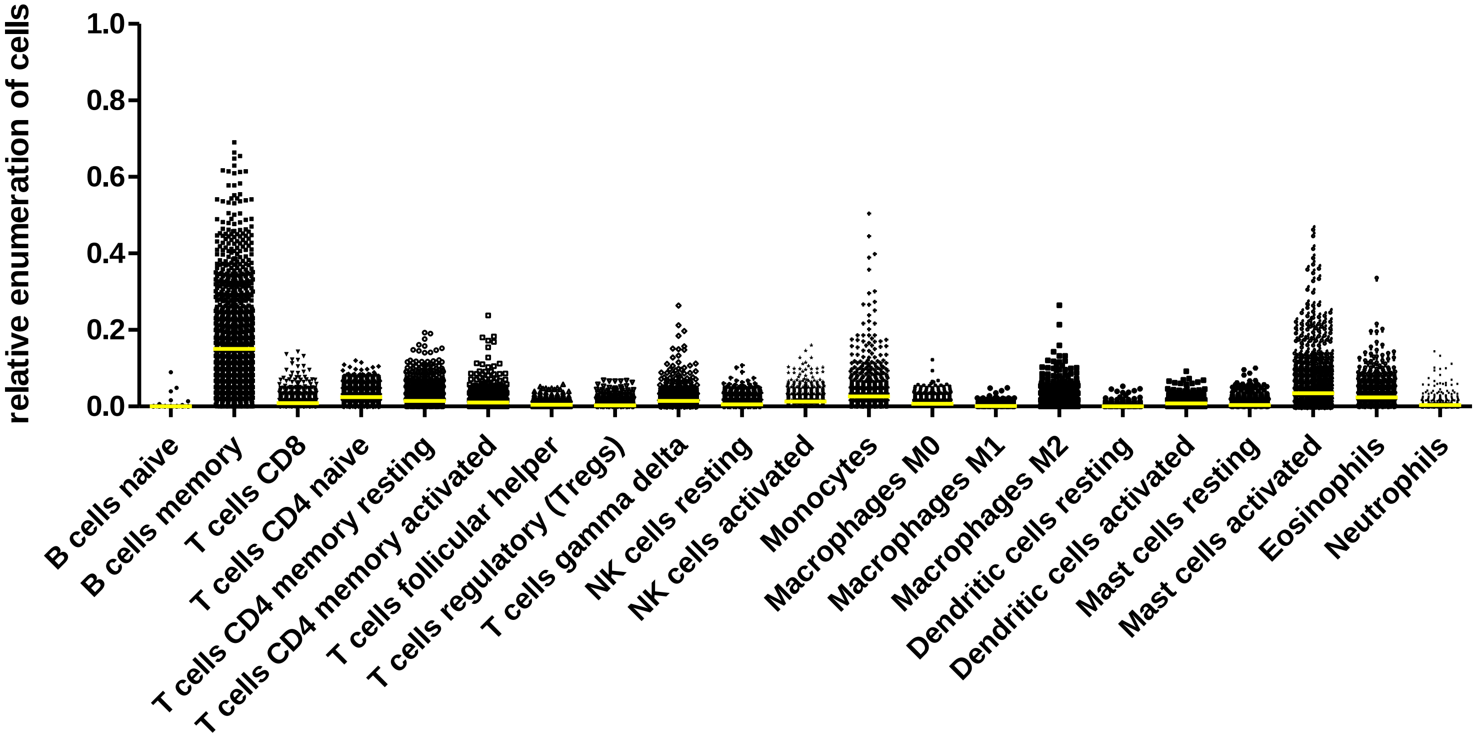

Supplementary Figure 2. FACS sorted plasma cells from bone marrow with 90% purity for CD138 contain significant amounts of immune cells

Multyple Myeloma panel (number indicates position of primary antibody):

1. CD8 (C8/114B, Sanbio, 1:200) – OPAL570; 2. VISTA (D1L2G, Cell Signaling Technologies, 1:100) – OPAL520; 3. CD11b (EP1345Y, Abcam, 1: 250) – OPAL620; 4. CD138 (B-A38, Cell Marque, 1:40) – OPAL690; 5. DAPI.

Immune suppressor panel (number indicates position of primary antibody):

1. CD4 (EP204, Cell Marque 1:100) – OPAL570; 2. CD8 (C8/144b, Sanbio, 1:200) – OPAL620; 3. Foxp3 (236A/E7, Abcam, 1:200) – OPAL650; 4. VISTA (D1L2G, Cell Signaling Technologies, 1:100) – OPAL520; 5. CD163 (MRQ26, Cell Marque, 1:50) – OPAL540; 6. CD11b (EP1345Y, Abcam, 1:200) – OPAL690; 7. DAPI.

Supplementary Table 1. Antibody panels used for immunofluorescence

OS analysis in 470 patients; [H65 (n=324) and H87 (n=146)]							
	OS univariate				OS multivariate		
	HR	[95%CI]	p value		HR	95%CI	p value
**VISTA**	0.72	[0.60-0.87]	6.19E-04		0.74	[0.56-0.85]	1.61E-03
**Purity of CD138+ cells**	1.02	[1.00-1.04]	0.054		1.01	[0.68-1.49]	0.959
**logodds(purity)**	1.14	[0.99-1.3]	0.0641		1.01	[0.96-1.08]	0.637

Supplementary Table 2. Purity of CD138+ plasma cells does not confound prognostic value of VISTA gene expression.

Treatment arm	Age	Sex	VISTA mRNA expression	Albumin [g/l]	Serum B2-microglobulin [mg/l]	1p-	1q(21) 1q	13q14-	17p13-	t(4;14)(p16;q32)	t(11;14)(q13;q32)	t(14;16)(q32;q23)	VISTA driven CD8 exclusion
MPT	M	76	10,5	21	6,8	no	no	yes	no	no	no	no	0,90
	F	78	10,4	36,3	2,8	no	no	no	no	no	no	no	1,22
	F	78	10,8	37	3,1	no	no	yes	yes	no	no	no	0,78
	M	81	10	29	6,2	no	no	no	no	no	no	no	1,25
	F	74	10,8	42,5	na								1,26
	F	69	8,6	28	5	no	no	no	no	no	no	no	0,74
	M	68	9,3	44,7	2,3	no	yes	yes	no	yes	no	no	0,88
	F	68	9,1	41	2,4			yes		no			2,29
	F	68	10,3	43	4,2			no	no	no	no	no	0,55
	F	71	9,8	30,4	4,8	no	yes	yes	no	yes	no	no	1,41
	M	71	9,8	28,5	3,8			no	no	no			1,30
MPR	F	70	11,1	36,4	2,3			no	no	no			1,50
	F	81	10,9	34	4,5	no	no	no	no	no	no	no	0,98
	F	73	11	31,5	na	no	no	no	no	no	no	no	1,09
	M	69	10,5	38,1	8,2		no	yes	no	no		no	1,42
	M	80	9,5	35,6	17,4	no	no	no	no	no	no	no	1,32
	F	75	10	36	5,4	no	no	yes	no	no	no	yes	1,35
	F	82	10,1	36,3	3,5			yes	no	yes			1,70
	M	71	11,6	50	12,7	no	no	no	no	no	no	no	1,43
	M	71	9,9	24	na		yes	yes					0,85
	M	69	12,3	36,1	7,6	no	yes	yes	no	no	no	no	1,33
	M	76	9,6	34	3,7	no	yes	yes	no	no	no	no	1,55

Supplementary Table 3. Characteristics of patients treated in the HOVOB87/NMSG18 trial, and whose tumors were used for in situ stainings.
MPT: melphalan, prednisolone and thalidomide; MPR: melphalan, prednisolone and lenalidomide.
